# Rheological and Physiological Consequences of Conversion of the Maternal Spiral Arteries for Uteroplacental Blood Flow during Human Pregnancy

**DOI:** 10.1016/j.placenta.2009.02.009

**Published:** 2009-06

**Authors:** G.J. Burton, A.W. Woods, E. Jauniaux, J.C.P. Kingdom

**Affiliations:** a Centre for Trophoblast Research, University of Cambridge, Cambridge, UK; b BP Institute for Multiphase Flow, University of Cambridge, Cambridge, UK; c Academic Department of Obstetrics and Gynaecology, Royal Free and University College, London, UK; d Department of Obstetrics and Gynecology, Mount Sinai Hospital, Toronto, Canada

**Keywords:** Spiral arteries, Uteroplacental circulation, Preeclampsia

## Abstract

Physiological conversion of the maternal spiral arteries is key to a successful human pregnancy. It involves loss of smooth muscle and the elastic lamina from the vessel wall as far as the inner third of the myometrium, and is associated with a 5–10-fold dilation at the vessel mouth. Failure of conversion accompanies common complications of pregnancy, such as early-onset preeclampsia and fetal growth restriction. Here, we model the effects of terminal dilation on inflow of blood into the placental intervillous space at term, using dimensions in the literature derived from three-dimensional reconstructions. We observe that dilation slows the rate of flow from 2 to 3 m/s in the non-dilated part of an artery of 0.4–0.5 mm diameter to approximately 10 cm/s at the 2.5 mm diameter mouth, depending on the exact radius and viscosity. This rate predicts a transit time through the intervillous space of approximately 25 s, which matches observed times closely. The model shows that in the absence of conversion blood will enter the intervillous space as a turbulent jet at rates of 1–2 m/s. We speculate that the high momentum will damage villous architecture, rupturing anchoring villi and creating echogenic cystic lesions as evidenced by ultrasound. The retention of smooth muscle will also increase the risk of spontaneous vasoconstriction and ischaemia–reperfusion injury, generating oxidative stress. Dilation has a surprisingly modest impact on total blood flow, and so we suggest the placental pathology associated with deficient conversion is dominated by rheological consequences rather than chronic hypoxia.

## 1 Introduction

Normal pregnancy demands that two distinct but inter-related changes in cardiovascular function take place in tandem. First, the blood supply to the uterus is enhanced and a maternal circulation to the placenta is established, effectively diverting blood away from the lower limbs. Second, several haemodynamic adjustments in the mother's circulation occur; her blood volume and cardiac output increase quickly by over one third, yet her blood pressure falls [1]. This apparent paradox is reconciled by a profound reduction in systemic vascular resistance until mid-gestation [2], along with a reduction in blood viscosity due largely to haemodilution. These adjustments are synergistic in promoting an effective uteroplacental blood supply.

A range of common complications of pregnancy, encompassing recurrent first and second trimester losses, fetal growth restriction (FGR), early-onset preeclampsia, spontaneous preterm labour and preterm premature rupture of the membranes, are associated with varying degrees of failure to anatomically transform the spiral arteries of the placental bed [3–6]. In addition, these complications are often associated with abnormal maternal adaptations to pregnancy in the second trimester, including failure to gain weight, lack of blood pressure reduction, and persistent non-pregnant haematocrit levels [7,8].

**Fig. 1:**
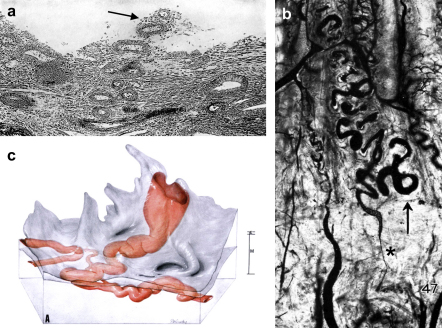
a) A photomicrograph of the human endometrium on the fourth day of menstruation showing an eroded spiral artery (arrowed) projecting freely into the uterine lumen. b) A photomicrograph of spiral arteries in a rhesus monkey during the phase of ovulation injected with India ink in gelatin. The arrow marks the endometrial–myometrial boundary, and a marked constriction (asterisked) can be seen in the spiral artery in the junctional zone just below. c) Reconstruction from serial sections of a converted spiral artery passing through the myometrium (M) and endometrium (E) before opening into the intervillous space through the basal plate of a term placenta. The widest dimension of the opening is given as 2.4 mm. Reproduced from Refs. [83], [15] and [16] respectively with permission of the Carnegie Institute of Washington.

Most of these pregnancy complications are unique to the human, and may relate to the fact that our invasive form of implantation, and subsequent haemochorial placentation, poses special haemodynamic challenges. These fall into two principal categories. Firstly, the spiral arteries of the human uterus perform contrasting roles during the menstrual cycle and in pregnancy. During the cycle they supply the endometrial stroma and the glands in preparation for implantation by the blastocyst, yet must retain the ability to contract as the upper layers of decidualized endometrium are shed during menstruation, in order to limit blood loss (Fig. 1a). Following implantation and embryogenesis, however, an inviolable supply is required to meet fetal demands, beginning in the second trimester but becoming critical towards the end of gestation due to fetal growth. Secondly, in the haemochorial situation the delicate fetal villi perfused by the low-pressure developing fetal circulation are immersed in maternal blood circulating at a potentially much higher pressure and velocity. It is essential, therefore, that the spiral arteries adapt so that they are capable of delivering large quantities of blood to the placental intervillous space, but at an appropriate rate and pressure. Conversion of the spiral arteries reconciles these various demands, and is key to a successful pregnancy.

Several recent publications have comprehensively reviewed the cellular mechanisms by which the spiral arteries undergo physiological conversion during early pregnancy [9–12]. Here, we consider the consequences of conversion in terms of the haemodynamics of intervillous blood flow. The principal aim is to clarify the roles that conversion serves, in order that we understand better the impact of failure of that process in pregnancies complicated by placentally related pathologies.

## 2 Anatomy of the uteroplacental circulation

### 2.1 Non-pregnant state

**Fig. 2:**
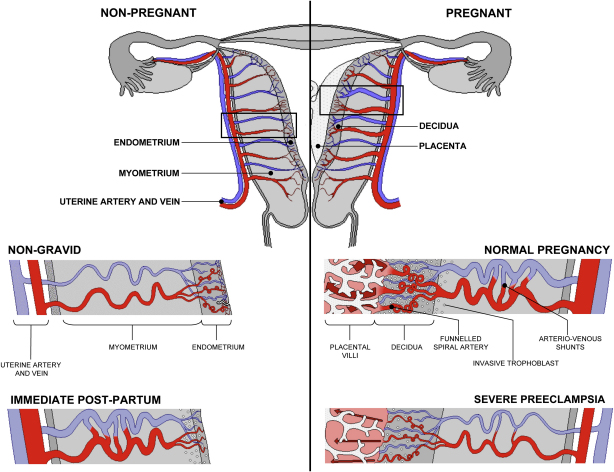
Diagrammatic representation of uterine and placental vasculature (red shading = arterial; blue shading = venous) in the non-pregnant, pregnant and immediate post-partum state. Normal pregnancy is characterized by the formation of large arterio-venous shunts that persist in the immediate post-partum period. By contrast pregnancies complicated by severe preeclampsia are characterized by minimal arterio-venous shunts, and thus narrower uterine arteries. Extravillous cytotrophoblast invasion in normal pregnancy (diamonds) extends beyond the decidua into the inner myometrium resulting in the formation of funnels at the discharging tips of the spiral arteries. Contrast with severe preeclampsia. (Prepared by Ms. Leslie Proctor, MSc.)

The uterus is supplied by the left and right uterine arteries, which ascend along the lateral aspect within the broad ligament (Fig. 2), and terminate by anastomosing with the respective ovarian artery. At intervals along their length the vessels give rise to arcuate arteries that pass medially and penetrate the myometrium. The arcuate arteries divide almost immediately into anterior and posterior branches that run circumferentially between the outer and middle thirds of the myometrium, and anastomose freely with their counterparts from the opposite side in the midline. During their course the arcuate arteries give rise to the radial arteries that are directed towards the lumen of the uterus. As they approach the myometrial–endometrial boundary each radial artery gives off lateral branches, the basal arteries that supply the myometrium and the deeper basalis parts of the endometrium, and continues as a spiral artery [11]. The spiral arteries are highly coiled within the basalis and the deeper parts of the functionalis, but as they approach the uterine lumen they suddenly narrow, and divide into several smaller branches that follow a straighter course before terminating in a capillary plexus just beneath the uterine epithelium. As it passes through the endometrium each spiral artery also gives off small branches supplying the capillary plexus surrounding the uterine glands.

In the non-pregnant state the walls of the spiral and radial arteries contain large quantities of smooth muscle equipped with a rich autonomic innervation. Hence, they are highly responsive to both exogenous and endogenous adrenergic stimuli [13,14]. The myometrial segment of the spiral artery just proximal to the myometrial–endometrial junction seems particularly important in this respect, as constrictions have been noted to be common at this point in both the human and the rhesus monkey (Fig. 1b) [14–16]. The inner myometrium is now recognised to be a specialised region, often referred to as the junctional zone [17]. Spontaneous vasoconstriction of the spiral artery in this region was observed by Markee to precede, and possibly induce, menstruation in the rhesus monkey, but also serves to limit blood loss from the disrupted distal end of the artery during the menses [18].

### 2.2 Changes in pregnancy

During early pregnancy endovascular extravillous trophoblast cells migrate down the lumens of the spiral arteries, while interstitial trophoblast cells migrate through the endometrial stroma and penetrate the vessel walls from their outside. In normal pregnancy, the interstitial trophoblast cells invade as deep as the inner third of the myometrium, where they progressively transform into immotile giant cells (Fig. 2). Both endovascular and interstitial invasion are associated with the physiological conversion of the spiral arteries, although the molecular mechanisms involved are still unclear [9,11]. During this process the arteries loose the smooth muscle in their walls and their elastic lamina, and as a result it is commonly stated that the vessels dilate and are converted into flaccid conduits. The extent of conversion varies across the placental bed, and is greatest in the central region where trophoblast invasion is most extensive [19].

**Fig. 3:**
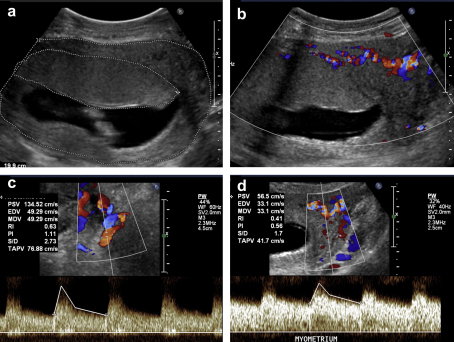
Representative real-time and colour/pulsed Doppler images of placental development at 12 weeks gestation. a) Transverse view of body of uterus outlining the uterine wall (outer line) with an anteriorly located placenta (inner line). b) The same view with colour Doppler (at conventional arterial setting of 38 cm/s). Note flow signals in this range are confined to the myometrium. c) Colour/pulsed Doppler identification of the proximal left uterine artery. Note low-impedance waveform with high (135 cm/s) peak systolic velocity. d) Colour-pulsed Doppler identification of arterial signals in the lateral myometrium, above the left proximal uterine artery. Note low-impedance waveform similar to Fig. 3c but at lower (56 cm/s) velocity. No such signals are observed entering the basal plate of the placenta. Whilst these images suggest intra-myometrial shunting at the end of the first trimester, the extent to which this phenomenon occurs in the second and third trimesters requires further research.

The final outcome was elegantly demonstrated by Harris and Ramsey, who performed three-dimensional reconstructions of a number of spiral arteries from hysterectomy specimens at various stages of pregnancy [16]. They observed that the arteries undergo a generalised, but non-uniform, dilation as pregnancy advances, with considerable variation in size between arteries within the same specimen, and even at different points along individual arteries. Most importantly, they found that the terminal coils of the spiral arteries are enormously dilated, often reaching 2–3 mm in diameter. This transition is quite abrupt, so that the dilated segments form a funnel-shaped chamber that opens through the basal plate, often with a slit-like orifice. Thus, they depicted the terminal part of a spiral artery at term having a maximum diameter of 2.4 mm (Fig. 1c), which represents an approximately 4-fold increase in the diameter of the vessel at the myometrial–endometrial boundary and within the endometrium. Why only the final section of a converted artery is involved in this exaggerated dilatation is not clear, but it may be sculpted by the extent of the endovascular invasion of the vessels in early pregnancy. Initially the invasion is so extensive that the tips of the arteries are effectively plugged by the trophoblast cells [14,20], and so there is little, if any, maternal blood flow into the placenta (Fig. 3).

Equally, it should be remembered that trophoblast invasion reaches neither the radial arteries, nor the arcuate and uterine arteries. Nonetheless, all these undergo profound dilation during pregnancy, particularly beneath the implantation site [21,22]. From unique radiographic studies Burchell observed that the diameter of the uterine artery doubles by 6.5 weeks of pregnancy. He also observed another unique property of the uterine circulation during pregnancy, namely that the diameter of the vessels increases, rather than decreases, as they approach their target organ. Thus, by mid-pregnancy the diameter of the arcuate arteries exceeds that of the uterine vessels, and by term some are twice the diameter [22]. This progressive dilation is reflected in sonographic measurements demonstrating that the peak systolic velocity and pulsatility index are higher in the uterine artery compared to the arcuate artery (Fig. 3) [23].

This non-trophoblast induced dilation is most likely a combined response to endocrine stimulation and nitric oxide-mediated flow-dilation signals, as uterine blood flow increases from approximately 45 ml/min in the follicular phase to approximately 750 ml/min at term [24,25]. The uterine arteries are responsive to oestrogens [26,27], and their endothelial and smooth muscle cells are also rich in receptors for human chorionic gonadotropin (hCG) [28]. These are particularly dense on both cell types in the smaller resistance branches of the artery, and a combination of *in vitro* and *in vivo* data confirms that administration of hCG causes vasodilation, mediated through increased production of eicosanoids [29]. Placental growth factor has also been shown to induce vasodilation of human endometrial resistance arteries isolated from the lower uterine segment during caesarean section [30]. This effect is mediated most likely through the VEGFR-1 receptor, and acts partially through increased nitric oxide production. Vascular endothelial growth factor itself is also a potent dilator of the uterine artery in sheep [31].

The loss of smooth muscle from the walls of the spiral arteries will indeed convert them into flaccid conduits. The normal extent of trophoblast invasion as far as the inner third of the myometrium means that the highly vasoreactive myometrial segment of the spiral arteries is included in the conversion process. Indeed, it is intriguing that trophoblast invasion stops at this level, suggesting the function it performs has been completed. The myometrial segment of a spiral artery is responsive to steroid hormones, and is substantially remodelled before and during pregnancy [17]. Nonetheless, some vasoreactivity does persist since narrowings, almost to the point of occlusion, have been outlined radiographically in this segment in the human at mid-pregnancy [32].

It is notable that trophoblast invasion does not penetrate as far in the rhesus monkey, being restricted to the endometrium [14]. In this species, spontaneous vasoconstriction of the myometrial spiral arteries may account for the radiographic findings that blood flow into the intervillous space from individual spiral arteries is intermittent, even during periods of uterine relaxation when myometrial activity and maternal blood pressure are constant [33]. Thus, while some arteries display continual inflow, others open and close on an apparently random basis. Furthermore, injection of epinephrine into the maternal circulation reduces both the number and size of the maternal arterial spurts observed in a dose-responsive manner [14]. The reduction is independent of uterine contractions, and with sufficient dosage all the spiral arteries can be constricted.

Equivalent studies have not been performed in the human, and so the extent to which intermittent perfusion of the intervillous space occurs in our species is uncertain. Radiographic images of contrast medium entering the intervillous space reveal the characteristic ‘doughnut rings’ in apparently random areas of the placenta, suggesting that it may be the case. We have also observed ultrasound evidence of brief periods of discontinuous flow [34].

Recently, a combination of sonography, vascular casting and oxygen measurements has shown that extensive shunting occurs within the myometrium under the placental bed [35]. Whether the formation of these shunts is related to trophoblast invasion is not clear, but they are not observed in the opposite wall of the uterus. Their presence means previous assumptions that the increased uterine artery blood flow in pregnancy is directed entirely into the intervillous space via the spiral arteries need to be challenged (Fig. 2). These shunts may be merely one example of how a healthy pregnant woman successfully reduces her systemic vascular resistance.

### 2.3 The intervillous circulation

From the mouth of a spiral artery the maternal blood is delivered into the villus-free central cavity of a lobule, as demonstrated by vascular casting [36], and then disperses radially between the villi. Lobules are variable in size depending on their position, with larger ones of a diameter up to 3 cm being located centrally in the disc. Moll and colleagues described the unique nature of the intervillous circulation in the haemochorial placenta [25,37], referring to the circulation as an open system in contrast to other circulatory beds of the systemic circulation where blood traverses from arteries via capillary beds to veins. Because the spiral arteries open into the intervillous space that essentially is a large lake of blood, there is little or no impedance to blood flow. These investigators considered the placenta to act as a large arterio-venous shunt, and whilst that remains true, the concept must now be extended to operate in parallel with the extensive shunts in the myometrium [35]. Others have likened the passage of the maternal blood between the villi to the percolation of fluid through a porous medium [38]. As the blood passes over the surface of the placental villi materno-fetal exchange takes place, including the diffusion of oxygen. This is evidenced by the fact that antioxidant enzyme activity is higher in villous tissues sampled from the centre of a lobule than in the periphery, indicating a gradient in the prevailing oxygen concentration [39].

## 3 Modelling flow in the spiral arteries and the discharge into the intervillous space

Given the substantial changes in the more proximal uterine vasculature during pregnancy, the unique conversion of the distal portions of the spiral arteries must have additional physiological advantages other than to add in a small way to the overall reduction in uteroplacental and systemic vascular resistance. In an attempt to answer this question, we have modelled flow in the spiral arteries at term, and will consider the impact of conversion on four aspects of flow into the intervillous space that are critical for placental exchange, namely the rate, pressure, constancy and volume of flow. We will then consider the impact on uterine arterial vascular resistance.

The spiral arteries are modelled as being 10 mm in length, with a pressure drop of 80 mmHg along their length. We have taken the total flow supplied to the uterus to be 750 ml/min [24], although as discussed previously it is now uncertain whether all this volume of maternal blood is delivered into the intervillous space. The volume flow rate *Q* through each artery is governed by the local Poiseuille flow relation

$$Q=\frac{\pi r^{4}}{8\mu }\frac{\mathrm{d}p}{\mathrm{d}x}$$

where *r*(*x*) is the local radius of the artery, with *x* the downstream distance, *p* the pressure and *μ* the viscosity of the blood. Here, we have assumed that the change of the radius of the artery over a distance comparable to the radius is small compared to the radius so that, to leading order, the flow is parallel to the downstream direction *x*.

**Fig. 4:**
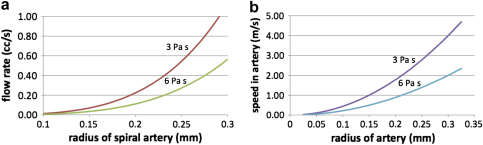
a) Graphs showing the relationship between the flow volume and the radius of a spiral artery, assuming a length of 1 cm and a pressure drop of 80 mmHg. The upper curves correspond to a viscosity of 3 mPa s and the lower curves to a viscosity of 6 mPa s. b) Graphs showing the relationship between the speed of flow and the radius of a spiral artery, assuming a constant radius and no dilation.

If the radius is constant along the length of the artery then the pressure gradient is constant, and in this case Fig. 4a illustrates the flow rate as a function of the radius. Curves are given for blood viscosities of 3 mPa s (upper curve) and 6 mPa s (lower curve), which span the measurements provided for the end of pregnancy [8]. We infer from this figure that for an artery with a radius in the order of 0.2–0.25 mm (diameter 0.4–0.5 mm), as depicted by Harris and Ramsey (Fig. 1c) [16], the flow rate will be about 0.2–0.4 ml/s. With smaller arteries, the flow rate decreases rapidly given the dependence of the flow on the fourth power of the radius. The mean flow speed in the artery is in the order of 1–2 m/s, given the flow rate and cross-sectional area, as indicated in Fig. 4b.

This simple calculation suggests that in the absence of any dilation at the end of the artery a very high speed jet, of 1–2 m/s depending on the viscosity and the exact radius, would enter the intervillous space, and this would have considerable momentum. Such a flow rate would imply a Reynolds number of the flow of about 20–80, and so the viscous stress will dominate the flow resistance.

**Fig. 5:**
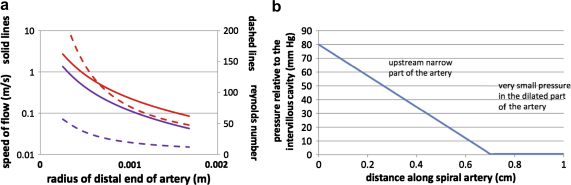
a) Graphs showing the relationship between speed of flow on exit from the artery (solid lines) and Reynolds number (dashed lines) with the radius of the artery. The upper curves correspond to a viscosity of 3 mPa s and the lower curves to a viscosity of 6 mPa s. Note that the scale for speed is logarithmic, so that dilation leads to a major reduction in exit velocity. b) Graph showing the pressure gradient along the non-dilated and dilated portions of a converted spiral artery.

However, if there is a region of dilation in the artery near the opening into the intervillous space, then the flow speed will decrease, and the overall flow for a given pressure gradient will increase. For example, in the simplified case that there is a linearly divergent section of the artery in the region *a* < *x* < *b*, where *x* = *b* is the terminal end of the artery, with the radius increasing according to *r* = *r*_0_ + *c*(*x* − *a*) in this terminal zone, from a fixed radius *r*_0_ upstream to a final radius *r*_0_ + *c*(*b* − *a*), then for a given pressure drop across the artery, the speed of the flow entering the intervillous space decreases substantially as the increase in radius becomes larger, as shown in the blue line in Fig. 5a. The flow has been calculated from the modified relation for the pressure drop as a function of the flow rate *Q*

$$\Delta P=\frac{8\mu Q}{\pi r_{0}^{4}}\left[a-\frac{r_{0}^{4}}{3c{\left(r_{0}+c\left(b-a\right)\right)}^{3}}+\frac{r_{0}}{3c}\right]$$

In turn, the Reynolds number of the jet, given by Re = ur/*ν* where *ν* is the kinematic viscosity of the blood, also decreases as it enters the intervillous space as the amount that the distal end of the artery diverges increases. In Fig. 5a, we assume the divergence occurs in the last 3 mm of the artery, with the radius increasing from an upstream value of 0.25–1.2 mm at the mouth as depicted by Harris and Ramsey [16]. The effect of the dilation is to reduce the flow speed substantially down to values in the region of 10 cm/s from the upstream values of 2–3 m/s, owing to the larger flow area.

It is notable that the volume of flow remains similar in both these cases, with the non-diverging artery of radius 0.25 mm supplying a flow of 0.27 ml/s, and that with the dilated end providing 0.37 ml/s in the present calculations. If we assume a flow of 0.2–0.4 ml/s and a total uterine blood flow of 750 ml/min, then we estimate that 30–60 spiral arteries are required to deliver that flow. This is towards the lower end of the range given by Boyd and Hamilton [40], but approximates closely to the estimate of 40–50 of Reynolds [41] based on the number of lobules observed. As Boyd and Hamilton comment, counts based on openings through the basal plate alone may overestimate the number of functional arteries. Remodelling during pregnancy can leave some segments of the arteries redundant.

Finally, it is of interest to note how the pressure varies along the spiral artery. It is seen that the pressure losses occur primarily upstream of the dilation zone, and in this distal segment the pressure is comparable to that of the intervillous space (Fig. 5b). This equates with the pressures measured by Moll et al. in the terminal parts of the spiral arteries in the rhesus monkey just before they enter into the intervillous space [37].

## 4 Physiological consequences of spiral arterial conversion

Although we recognise the values derived from these calculations are approximations due to assumptions inherent in the model, nonetheless they allow us to appreciate the physiological consequences dependent on the terminal dilation. These will now be considered in association with the other changes in arterial structure associated with conversion.

### 4.1 Rate of inflow

**Fig. 6:**
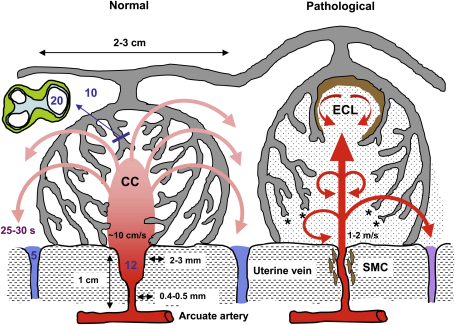
Diagrammatic representation (not to scale) of the effects of spiral artery conversion on the inflow of maternal blood into the intervillous space and on lobule architecture predicted by modelling. Dilation of the distal segment in normal pregnancies will reduce the velocity of incoming blood, and the residual momentum will carry the blood into the central cavity (CC) of the lobule, from where it will disperse evenly through the villous tree. Transit time to the uterine vein is estimated to be in the order of 25–30 s, allowing adequate time for oxygen exchange. The pressure of the maternal blood, indicated in mmHg by the figures in blue, will drop across the non-dilated segment of the spiral artery, the dimensions of which are given alongside. In pathological pregnancies, where no or very limited conversion occurs, the maternal blood will enter the intervillous space at speeds of 1–2 m/s. The high Reynolds number predicts turbulent flow, indicated by the circular arrows. We suggest that the high momentum ruptures anchoring villi (asterisked) and displaces others to form echogenic cystic lesions (ECL) lined by thrombus (brown). The transit time will be reduced, so that oxygen exchange is impaired and blood leaves in the uterine vein with a higher oxygen concentration than normal. Trophoblastic microparticulate debris (dotted) may be dislodged from the villous surface, leading to maternal endothelial cell activation. Finally, the retention of smooth muscle cells (SMC) around the spiral artery will increase the risk of spontaneous vasoconstriction and ischaemia–reperfusion injury.

The potential impact of the jets of maternal blood emerging from the arteries within the intervillous space depends on the distance they travel prior to meeting other tissues. Jets emerging from arteries with a diameter of 0.4 mm at a speed in the order of 1.0 m/s will tend to mix and entrain blood within the intervillous space, but may require distances of a few millimetres to centimetres to decelerate to speeds in the order of 10 cm/s. In contrast, the slower flow from the dilated arteries will already emerge at these lower speeds, and will continue to slow as it moves out. Therefore, the potential damage of the momentum flux in the inflowing blood will be considerably smaller if the distal end of the artery dilates (Fig. 6). Reynolds suggested that it is the force of the maternal arterial spurts that shapes the placental lobules towards the end of the first trimester and forms the central cavities [41]. However, if the incoming jet has too high a velocity it may create villous damage as will be discussed later.

It is also of interest to estimate the time of travel through the villous meshwork comprising the wall of a lobule before the blood drains into the openings of the uterine veins. Typically, lobules have a diameter of 2–3 cm, and so assuming they take the shape of a hemisphere, and the incoming flow passes uniformly through the lobule, then the travel time with a flow of order 0.2–0.4 ml/s is about 10–20 s. This fits closely with the value of 25 s for the clearance of radiopaque dye from the intervillous space at term [22]. The terminal villi have diameters in the order of 0.1 mm, and the intervillous pores between them are of a similar scale. The diffusion of oxygen through the plasma over distances of this scale requires a time in the order of 5 s, assuming a diffusion coefficient of 1e−9 m^2^/s. Therefore, the normal rate of flow through the lobule provides sufficient time for oxygen exchange, and this may set the scale for the lobules.

### 4.2 Pressure of inflow

For effective diffusional exchange a thin barrier between the maternal and fetal circulations needs to be maintained. In the human, this is facilitated by the formation of vasculosyncytial membranes where dilated fetal capillaries obtrude from the surface of the villus, stretching the trophoblast covering and causing local thinning [42]. In the haemochorial situation it is therefore essential that the pressure in the intervillous space is lower than that in the fetal capillaries, if compression of the latter is to be avoided. The restriction of fetal blood flow by external compression has been referred to as the ‘sluice flow’ phenomenon [43], and the architecture of the villous haemochorial placenta would seem to make it particularly vulnerable to this effect [25]. Most fetal red cells are nucleated during the first two months after conception, resulting in a high blood viscosity and very high resistance to flow that is observed *in vivo* until around 14 weeks of gestation [44]. A high pressure inside the intervillous space would therefore prevent the establishment of a feto-placental circulation. Although, there is morphological evidence of a preferential feto-yolk sac circulation during the first two months of pregnancy, there is also *in vivo* evidence of a continuous systolic umbilical circulation from 7 weeks of gestation [45].

Experiments in which the maternal and fetal circulations of term placentas were pressurised to different levels revealed that the critical determinant of fetal capillary size, and hence mean membrane thickness, is the pressure differential between the two circulations, whatever the absolute values might be [46]. Evidence that this principle holds *in vivo* comes from analysis of the effects of maternal posture on umbilical arterial waveforms. In the supine position the pregnant uterus compresses the inferior vena cava, leading to a build-up in pressure and a reduction in intervillous blood flow [47,48]. At the same time there is a reversible increase in the umbilical arterial resistance, indicating acute compression of the fetal placental vessels [49]. It is essential therefore that the transmembrane pressure differential is positive in a fetal to maternal direction if the fetal capillaries are to remain distended. As can be seen in Fig. 5b most of the maternal pressure drop occurs across the non-dilated part of the artery, so that the pressure in the terminal dilated section of the artery is equivalent to that in the intervillous space.

### 4.3 Constancy of blood flow

The effects on rate and pressure of inflow are mediated by the terminal dilations of the spiral arteries, yet the vessels undergo conversion as far as the inner third of the myometrium where the vessel does not dilate to the same extent. As discussed earlier, we speculate that the goal of this deeper invasion is to remove the smooth muscle from the highly contractile segment of the spiral artery in the junctional zone (Fig. 1b). Doing so will reduce the risk of spontaneous vasoconstriction, and so the extent of intermittent perfusion that might otherwise occur, as evinced by the rhesus monkey. Ensuring an uninterruptible circulation to the placenta must be of the utmost importance, since removal of the segment that normally prevents excessive menstrual blood loss might reasonably be expected to predispose the woman to an increased risk of post-partum haemorrhage. Although experiments to examine the effects of administration of catecholamines on uteroplacental perfusion cannot be performed in the human, it is notable that patients with high levels of endogenous release through phaeochromocytoma have an elevated fetal loss rate in the second and third trimesters of pregnancy, which may be due to excessive constriction of these segments [50].

For much of pregnancy intermittent placental perfusion may be of little consequence, as the maternal blood retained within the intervillous space will provide a supply of oxygen and nutrients to tide the placental tissues over until the circulation restarts. However, towards term, when fetal and placental oxygen extraction are at their maximum, even temporary cessation in the circulation is likely to lead to a dip in the oxygen concentration within the intervillous space. This will be exacerbated by the fact that the volume of maternal blood acting as a reservoir around each villus will decrease as the size of the intervillous pores diminishes [51]. Fluctuating oxygen concentrations is a powerful inducer of placental oxidative stress [52], and so any mismatch in maternal supply and feto-placental demand may have significant physiological consequences.

Physiological conversion may therefore be a means by which the conflicting requirements for vasoreactivity to support menstruation, and vaso-inactivity to support placental perfusion, can be met within a single vessel.

### 4.4 Volume of intervillous blood flow

Uterine artery blood flow increases dramatically during gestation in all mammalian species whether or not trophoblast invasion occurs, and so is most likely an endocrine mediated event. The dilatation of the human uterine artery initially observed by Burchell will be more than adequate to accommodate the increase in uteroplacental flow measured during pregnancy [22,24]. As shown above, dilating the distal segment of a spiral artery has a relatively modest effect on the total volume of flow, as the proximal unconverted segment of the parent radial artery will always be rate-limiting.

### 4.5 Uteroplacental vascular resistance

Dilation of the distal segment of a spiral artery will reduce the resistance to flow within the vessel. One of the few studies to test for correlation between Doppler assessments of uterine arterial resistance and morphological quantification of spiral arterial trophoblast invasion in first trimester pregnancies found that low-resistance was associated with a greater proportion of the arteries displaying endovascular trophoblast [53]. Equally, both the resistance index and the pulsatility index have been reported to be lower in spiral arteries in the central region of the placental bed during the second trimester than in peripheral regions [54], and these findings correlate closely with the normal pattern of extravillous trophoblast invasion [55]. However, a more recent study found no difference between the two regions [56]. Any reduction in resistance will increase flow, and potentially lead to further dilation of the artery through increased shear stress and nitric oxide-mediated pathways in a feed-forward fashion. However, the relative contribution of this effect compared to other major changes occurring during early pregnancy, such as unplugging of the spiral arteries allowing free flow of blood into the intervillous space [57,58], is difficult to estimate.

The recent evidence of Schaaps et al., who observed that the resistance index in the uterine artery did not change following delivery of the placenta, indicates that flow through the spiral arteries and the intervillous space has little impact on overall resistance [35]. In addition, although there is usually a good correlation between uterine artery waveforms and the incidence of physiological conversion [59,60], this is not universal. Thus, Aardema et al. recorded normal waveforms in 14 normal pregnancies, of which 5 were found to have absent physiological conversion on biopsy [61].

These data suggest that uterine artery vascular resistance may be a proxy marker for trophoblast invasion, but that is not determined by it. Other factors, such as the release of vasoreactive mediators, or inward eutrophic remodelling of the radial arteries, may be more directly associated with uterine artery resistance [53,62].

### 4.6 Overview

Reviewing these data it is clear that physiological conversion of the spiral arteries has little impact on the volume of maternal blood flowing into the placenta. In all species this is likely to be mediated by endocrine factors acting on the whole vascular arcade from the uterine arteries onwards, and on maternal blood viscosity and erythrocyte deformability. However, conversion will have profound effects on the rate of delivery, and on the constancy of the maternal blood flow. Ramsey et al. put this succinctly when they proposed that the terminal dilatations formed an antechamber to the intervillous space in which the velocity and force of the maternal blood is reduced [63]. In epitheliochorial and endotheliochorial placentas the maternal blood is retained within a vascular network, with resistance arterioles present between the arteries and the maternal placental capillary bed. The same problems do not arise in this configuration, and this may explain why trophoblast invasion and physiological conversion of the arteries are not seen in species with these placentas.

## 5 Pathological correlates of deficient spiral arterial conversion

It is well established that defective trophoblast invasion and a failure to convert the spiral arteries is associated with early-onset preeclampsia and FGR [11,17,64,65]. Quantitative studies are difficult to interpret because in most cases investigators are working with small biopsy samples that may not accurately reflect changes elsewhere in the placental bed [12]. Nonetheless, it appears that there is a gradation between the normal and the preeclamptic state, and that in both normal and preeclamptic cases the most extensively converted vessels are to be found in the centre of the placental bed [5,19]. Defective invasion and failure to convert the arteries has also been associated with spontaneous miscarriage and premature rupture of the membranes [4,6,66]. On the basis of the preceding analysis, what might the consequences of failed conversion be for placental perfusion? When considering these, the effects of deficient conversion must be clearly separated from the secondary pathology in the vessels that is often superimposed.

Firstly, the maternal blood will enter the intervillous space at a greater velocity than normal, the calculations above indicating in excess of 1 m/s (Fig. 6). In the past, we have termed this the ‘hose effect’ [67], and on ultrasound the inflow appears as jet-like streams surrounded by turbulence. The force is sufficient to drive apart the villous branches and form intervillous lakes (also called maternal lakes). In contrast to the physiological central cavities of lobules these lakes are often lined by thrombotic material, consistent with the pattern of turbulent inflow. These have been termed echogenic cystic lesions (ECL) and are of prognostic significance when uterine artery Doppler is abnormal [68].

In the most severe cases of deficient arterial conversion the intervillous circulation is abnormal from the beginning of the second trimester, resulting in pronounced ultrasonographic changes in texture that have led the organ to be described *in vivo* as “jelly-like” or ‘wobbly’ [67,69]. This refers to the overall appearance of the placenta which has its chorionic plate pushed up by jet-like streams. These gross morphological changes can be identified on ultrasound as early as 14–15 weeks of gestation [69]. They are thought to arise from reduced numbers of, or rupture of remaining, anchoring villi (Fig. 6). Rupture of the anchoring villi will have a profound effect on placental architecture, but will also interrupt the supply of extravillous trophoblast, impairing further arterial modification.

In addition, even if blood entering at 1.0 m/s dilutes by a factor of 10–100 as it moves through a distance of a few millimetres to a centimetres, it will traverse much of the lobule in the order of 1 s as it decelerates and mixes. As a result, materno-fetal oxygen exchange will be impaired, even though the total volume of blood being supplied to the intervillous space from the arteries is comparable to that from the dilated arteries. This could explain the higher oxygen content in the uterine venous blood reported in cases of FGR [70]. These high velocities may also damage the villous surface at the microscopic level, causing the release of syncytial sprouts and trophoblast fragments into the maternal circulation that might then stimulate a maternal inflammatory response [71,72].

Secondly, it is likely that defective trophoblastic invasion will lead to a greater degree of intermittent perfusion of the intervillous space as described above. There is general consensus that it is the myometrial segments of the spiral arteries that are most affected in early-onset preeclampsia and FGR [3]. Consequently, one would expect the contractile segments to be retained, which will predispose the placenta to hypoxia–reoxygenation. This is a potent inducer of placental oxidative stress *in vitro*, and of many of the changes seen in early-onset preeclampsia [73–75].

Reduced trophoblast invasion and deficient conversion are associated with increased impedance within the spiral arteries in cases of early-onset preeclampsia, although interestingly the central/peripheral differential is still maintained [76]. The resistance index in the uterine arteries is also increased in these patients, although this may reflect changes in the walls of the radial arteries more than in the distal parts of the spiral arteries [62]. If so, then the association between abnormal uterine artery Doppler and severe early-onset preeclampsia may be as a result of high systemic vascular resistance, as opposed to a defect in delivery of maternal blood to the intervillous space.

It seems likely that failure of conversion of the spiral arteries by itself will not influence the volume of maternal blood flow to the placenta, for this will be determined by upstream changes in the rate-limiting radial and arcuate arteries. Thus, there is little basis to assume that the placenta will be hypoxic, and indeed there is no metabolic or other evidence to support this common assumption in cases of preeclampsia [77,78]. However, secondary pathology, such as acute atherosis and thrombosis does occur within the radial and spiral arteries, and has been extensively documented in preeclamptic pregnancies [11,17,79]. These secondary changes are likely to have a major impact on intervillous blood flow for they lead to a significant reduction in the calibre of the vessels, at least in non-perfused biopsy samples. This may explain why placental villous infarction correlates with decidual vasculopathy in the placental bed and abnormal uterine artery Doppler, rather than with thrombophilic defects in the mother [68,69]. Atherotic lesions could thus account for the reduced placental perfusion reported in cases of early-onset preeclampsia [48,80]. Despite this potential restriction to flow, no correlation was found between the incidence of such lesions and the uterine artery waveform [61].

## 6 Remaining questions

What becomes clear from this review is that flow through the spiral arteries appears to play no role in determining the uterine arterial waveform in either normal or pathological pregnancies. Why then is there usually an association between the degree of conversion and Doppler indices of resistance to blood flow such as the pulsatility index? If we accept that endocrine mediated dilation of the radial and arcuate arteries, and the formation of myometrial arterio-venous anastomoses are the major determinants of vascular resistance in the uteroplacental circulation, then it is possible to speculate as to how the two may be linked. It is becoming increasingly clear that some form of preconditioning of the spiral arteries precedes trophoblast invasion of the vessel walls, and this may well be endocrine mediated [10,11,17,81,82]. Deficient conversion may therefore reflect either an inadequate preconditioning signal or a lack of responsiveness of the cells in the vessel wall to that stimulus, rather than reduced trophoblast invasion as previously thought. In either case, if the same principle applies to the more proximal parts of the vascular network then deficient conversion may be associated with reduced dilation of the arcuate and radial arteries, and hence increased resistance, through a common mechanism.

Further research is also required to determine the morphological correlates of vascular resistance within the uteroplacental vasculature, and to investigate, for example using gadolinium-enhanced MRI, if myometrial arterio-venous anastomoses are less frequent or sizeable in pathological pregnancies with abnormal uterine artery Doppler. However, it is clear that when considering the impact of deficient conversion of the spiral arteries by itself on intervillous blood flow we should be thinking in terms of the velocity, pressure and constancy of the maternal inflow, and bear in mind that there may be associated secondary changes that have a greater impact on the volume of that flow.
